# Random Genetic Drift and Selective Pressures Shaping the *Blattabacterium* Genome

**DOI:** 10.1038/s41598-018-31796-6

**Published:** 2018-09-07

**Authors:** Austin Alleman, Kate L. Hertweck, Srini Kambhampati

**Affiliations:** 1 Department of Biology, University of Texas at Tyler, 3900 University Blvd., Tyler, Texas 75799 United States; 2 Present Address: Institute of Organismic and Molecular Evolution, Johannes Gutenberg University Mainz, Johannes von Müller Weg 6, Mainz, 55128 Germany

## Abstract

Estimates suggest that at least half of all extant insect genera harbor obligate bacterial mutualists. Whereas an endosymbiotic relationship imparts many benefits upon host and symbiont alike, the intracellular lifestyle has profound effects on the bacterial genome. The obligate endosymbiont genome is a product of opposing forces: genes important to host survival are maintained through physiological constraint, contrasted by the fixation of deleterious mutations and genome erosion through random genetic drift. The obligate cockroach endosymbiont, *Blattabacterium* – providing nutritional augmentation to its host in the form of amino acid synthesis – displays radical genome alterations when compared to its most recent free-living relative *Flavobacterium*. To date, eight *Blattabacterium* genomes have been published, affording an unparalleled opportunity to examine the direction and magnitude of selective forces acting upon this group of symbionts. Here, we find that the *Blattabacterium* genome is experiencing a 10-fold increase in selection rate compared to *Flavobacteria*. Additionally, the proportion of selection events is largely negative in direction, with only a handful of loci exhibiting signatures of positive selection. These findings suggest that the *Blattabacterium* genome will continue to erode, potentially resulting in an endosymbiont with an even further reduced genome, as seen in other insect groups such as Hemiptera.

## Introduction

Comprised of over one million species, Class *Insecta* is the most speciose group among animals; at least half of extant genera are estimated to harbor obligate bacterial mutualists^1–3^. While some intracellular bacteria can be harmful or even lethal to their insect host, many others play an important role in host survival and fecundity^3–8^. These primary bacterial symbionts exist obligately within the cells of the insect, and are often required for the survival and reproduction of their host organism^1,7–9^. An intercellular lifestyle affords endosymbiotic bacteria relative safety from competition and exploitation, in exchange for increased ecological flexibility imparted onto the host species. In many cases, these obligate bacterial mutualists function in the provisioning, recycling, or degradation of essential nutrients, and are vital to those insect species that subsist on nutritionally narrow diets, such as those composed primarily of woody material, plant sap, mammalian blood, or decaying organic material^8,10,11^. However, within some insect species primary bacterial endosymbionts also function in non-nutritional roles such as parasitoid defense^12^.

With the exception of a single cave-dwelling genus, *Noticola* (Blattodea, Nocticolidae), all cockroach species contain endosymbiotic bacteria within their fat bodies^1,5,13,14^. These obligate endosymbionts belong to the genus *Blattabacterium* (Class Flavobacteria, Phylum Bacteriodetes)^15,16^. Phylogenetic reconstruction suggests that cockroaches acquired these endosymbionts in a single infection event, dating between 300 million years ago - the approximate age of the first fossil roaches from the Carboniferous - and 140 million years ago, when currently extant families last shared a common ancestor^17,18^. Initially, the function of these endosymbionts was subject to speculation, owing to their recalcitrance to culture outside their host. However, modern DNA-sequencing techniques have allowed for the study of a number of *Blattabacterium* genomes. From these genomes, it was discovered that the function of *Blattabacterium* is primarily the synthesis of amino acids and vitamins from the nitrogenous waste products of the cockroach host^16,19^. Cockroaches store excess nitrogen as uric acid within their fat body cells^20^. The decaying organic matter on which cockroaches typically feed is poor in nitrogen content. Thus, a mechanism for recycling nitrogenous waste would be beneficial to any organism whose diet is nitrogen-deficient. Unlike most insects, which excrete waste nitrogen as uric acid, cockroaches excrete ammonia^21^. *Blattabacterium* are capable of utilizing both urea and ammonia because they contain an active urease as well as a functioning urea cycle that converts host urea to ammonia^22–24^. In addition, increases in dietary nitrogen intake by host cockroaches correlates with increases in uric acid buildup within that host’s fat bodies^20,21,23^.

Cockroaches represent an evolutionary lineage consisting of diverse and ancient taxa that have adapted to many habitats and exhibit broad nutritional ecology; their endosymbionts, therefore, represent an excellent system in which to assess relationships between these traits. To date, eight *Blattabacterium* genomes have been sequenced from the following cockroach host species: *Periplaneta americana*^19^, *Blatta germanica*^25^, *Cryptocercus punctulatus*^26^*, Blaberus giganteus*^27^, *Blatta orientalis*^28^, *Panesthia angustipennis*^29^, *Nauphoeta cinerea*^30^ and the termite, *Mastotermes darwiniensis*^31^. While these genomes share similar gene composition and genome architecture, each also displays unique capacities for metabolic and physiological function. Thus, while the results of phylogenetic analysis support the hypothesis of co-cladogenesis between the endosymbionts and hosts^17,18^, gene composition of *Blattabacterium* is not directly congruent with host phylogeny; rather it varies likely as a function of host nutrition, its relative importance in the mutualism, and the interaction between phenotypic constraint, environmental natural selection, and genetic drift.

An intracellular lifestyle strongly influences the selective pressures and evolutionary trajectories of bacterial endosymbionts^32^. Evolution of the bacterial endosymbiont genome is characterized by elevated mutation rates and biases resulting from the combined effects of physiological constraint preserving symbiont-critical genes, random genetic drift – driven by frequent population bottlenecks, bacterial asexuality, and lack of genetic recombination – and environmental selection acting to reduce genome size^18,28,33–47^. Endosymbionts have been shown to have higher substitution rates and values of non-synonymous to synonymous substitution rates a result attributed to small *N*_*e*_.^48,49^. Acting through Muller’s Ratchet, asexual reproduction can prevent the recovery of wild-type genotypes through recombination^50^. Loss of recombination is a result of lost DNA repair, uptake, and recombination genes; which is a common pattern of all sequenced bacterial endosymbionts [^51–56^ reviewed in ref.^57^].

However, selection in the form of physiological constraint acts to maintain genes important to the bacterial-insect symbiosis, although its role in the continued erosion of non-essential genes in the bacterial genome is largely unknown^45,58,59^. Described genomes from endosymbionts suggest that physiological constraint acts to maintain a gene set that retains its functionality for the host; though selection might also be driving the erosion of bacterial endosymbiont genomes. Certain metabolites ordinarily produced by the bacteria itself may now be obtained directly from the host; under such circumstances, these genes become superfluous and are necessary for neither bacterial survival nor continued host fecundity. As such, a smaller genome results in a cell that is faster and more efficient to reproduce. Particularly at the beginning of endosymbiosis, rapid loss of unnecessary genes^32^ may be advantageous [Reviewed in ref.^60^]. Thus, while random genetic drift does act to reduce the bacterial endosymbiont genome through Muller’s Ratchet, physiological constraint acts to preserve genes crucial to symbiosis while environmental selection favors a reduced, more energy-efficient genome.

When compared to free-living bacteria, endosymbionts exhibit increased levels of mutation at synonymous and non-synonymous sites, as well as higher d_N_/d_S_ ratios, indicating an increase in positive selective pressures and rapid protein evolution^33,61–63^. Thus, we may conclude that the endosymbiont genome is the result of interplay between random genetic drift and the reduction of genes through relaxed selection within large portions of the genome, and physiological constraint acting to preserve those genes vital to host survival and fecundity.

Genome evolution in insect endosymbionts has been the topic of a number of studies. Full genomes from several endosymbionts have been published, including *Buchnera aphidicola*^64^ from aphids, *Wigglesworthia*^65,66^ from the tsetse fly, *Blochmannia*^67^ from carpenter ants, and *Blattabacterium*^19^, from cockroaches. However, comparatively few of these genomes have been examined for signals of positive selection. Eight fully sequenced *Blattabacterium* genomes, in addition to the five fully-annotated free-living *Flavobacterium* genomes for comparison, offers a unique opportunity to investigate the patterns and processes that drive endosymbiotic genome evolution.

We estimated the positive and negative selection events in the genomes of all sequenced *Blattabacterium* strains, and compared them to those present within the closely-related^68^ but free-living *Flavobacterium* species (*F. indicum*, *F. johnsoniae*, and *F. psychrophilim*), to examine the similarities and differences between these two evolutionarily related, but divergent, groups. We hypothesized that patterns of selection acting upon the *Blattabacterium* genome will manifest as an elevation in both non-synonymous and synonymous mutation events, as well as a higher d_N_/d_S_ ratio at sites under significant levels of selection than the free-living *Flavobacterium -* indicating increased positive selection pressures and an elevated rate of protein evolution^63,69,70^. Additionally, we sought to determine whether patterns of selection observed in previous studies across limited numbers of genes are effective at predicting patterns of selection across an entire endosymbiont genome.

## Materials and Methods

### Sequence data

Homologous genes for eight *Blattabacterium* and five *Flavobacterium* species were manually compiled from genomes available in GenBank (Table 1). With *Blattabacterium* sp. *Cryptocercus punctulatus* as the model genome (as it is the smallest *Blattabacterium* genome and thus frames the “core” gene set of *Blattabacterium* and *Flavobacterium*) we manually BLASTed each loci against all other *Blattabacterium* genomes, as well as against existing *Flavobacterium* genomes. Resulting BLAST hits were then manually compiled into single homologue files in nucleotide fasta format. We applied Clusters of Orthologous Groups (COGs) to categorize the function of genes in our dataset. Given that genome-wide COG composition is very similar among *Blattabacterium*^30^, we assessed composition genome-wide as well as in the subset of homologous genes found in all taxa using the Bacterial Annotation System (BASys^71^).

**Table 1:** GenBank accession numbers for bacterial genomes used within this study.

Blattabacterium	From host species:	Host Family
**Accession Number**		
NC_017924.1	*Blaberus giganteus*	Blaberidae
NC_020195.1	*Blatta orientalis*	Blattidae
NC_013454.1	*Blatella germanica*	Ectobiidae
NC_016146.1	*Mastotermes darwiniensis*	Mastotermitidae
NC_022550.1	*Nauphoeta cinerea*	Blaberidae
NC_020510.1	*Panesthia angustipennis spadica*	Blaberidae
NC_013418.2	*Periplaneta americana*	Blattidae
NC_016621.1	*Cryptocercus punctulatus*	Cryptoceridae
**Flavobacterium**	**Species**	
NC_017025.1	*Flavobacterium indicum*	
NC_009441.1	*Flavobacterium johnsoniae*	
NC_009613.3	*Flavobacterium psychrophilum*	
NC_016510.2	*Flavobacterium columnare*	
NC_016001.1	*Flavobacterium branchiophilum*	
**Escherichia coli**	**Strain**	
CP002797.2	*Escherichia coli* NA114	
CP006784.1	*Escherichia coli* JJ1886	
CP002212.1	*Escherichia coli* str. Clone D i14	
CP002211.1	*Escherichia coli* str. Clone D i2	
AP009240.1	*Escherichia coli* SE11	
AE014075.1	*Escherichia coli* CFT073	
CP002729.1	*Escherichia coli* UMNK88	
CP002167.1	*Escherichia coli* UM146	

### Trimming, Alignment of Homologs, and Phylogeny Building

All scripts developed for this analysis (pre-processing, alignment, phylogenetics, and tests for selection) can be found at https://github.com/k8hertweck/Blattabacteria. Phylogenetic reconstruction of the evolutionary relationships of the eight *Blattabacterium* and five *Flavobacterium* species (with eight *Escherichia coli* strains as outgroup) was carried out using PhyloPhlan^72^ under default parameters and whole genomes obtained from GenBank (Table 1). For each set of homologous genes, the last three base pairs (e.g., the stop codon) of each sequence were removed using Prinseq^73^ and each homolog group was then aligned using TranslatorX^71^. Gaps present in more than 10% of an alignment were removed using trimAl and alignments summarized using readAl^74^. The best fitting model of molecular evolution for gene alignments, as assessed by both AIC and BIC in jModelTest2^75^ under default parameters, was GTR + G. A maximum likelihood tree for each homologous gene was calculated using this model in PhyML (www.atgc-montpellier.fr/phyml)^76^ and assessed using 100 bootstrap replicates (alternative models of evolution did not significantly affect tree topology, data not shown). The maximum likelihood tree for each gene (except *mia*, which possessed two gene copies for some taxa) was used to create a reconciled species tree using ASTRAL v4.7.8 (github.com/smirarab/ASTRAL/)^77^ and 100 bootstrap replicates.

### Selection Analysis

Selection analysis was performed using the HyPhy v2.2.1 (github.com/veg/hyphy)^78^ suite of programs. For this analysis, three different selection tests were used: HyPhy’s BUSTED^79^, Quick Selection Detection (implementing MEME [Mixed Effects Model of Evolution])^80^, and Branch Site REL^81^. Each of these programs used the same sets of input data; namely the HyPhy alignment combined with the PhyML tree for each gene. Parameters used for these tests may be found here: github.com/k8hertweck/Blattabacteria/blob/master/blattabacteriaBUSTED.bf, github.com/k8hertweck/Blattabacteria/blob/master/blattabacteriaQSD.bf, github.com/k8hertweck/Blattabacteria/blob/master/blattabacteriaBranchSiteREL.bf.

Summary statistics for all selection analyses performed were produced using an in-house script that may be found here: github.com/k8hertweck/Blattabacteria/blob/master/blattabacteriaSelectionSummary.sh. Summary statistics and input and output files for selection analysis may be found at: github.com/k8hertweck/Blattabacteria/tree/master/analysis. Statistics of particular import are those referencing branches under positive selection. This information was drawn from the BUSTED and Branch Sire REL output. Additional statistics were obtained from the output of blattabacteriaGeneSummary.sh (see Methods section ‘Trimming, Alignment of Homologs, and Phylogeny Building).

### Statistical Analyses

For analyzing the number of positive and negative sites of selection per gene length, a Linear Model was implemented using R version 3.4.4^82^. Within these models, number of positive and negative selection sites were log transformed. A handful of outliers were noted but retained, as their inclusion had a non-significant impact upon the resulting models.

Number of positive and negative selection events vs. individual COG size were carried out using Generalized Linear Models with Poisson distribution with number of selection sites as the response variable and total number of nucleotides in the genome associated with a specific COG as the explanatory variable. As COG and gene length analyses used different datasets - number of selection events per total number of nucleotides of all genes associated with a given COG and number of selection events by gene length, respectively - we found that differing models better fit each type of analysis.

## Results and Discussion

### Selection by Gene Length and COG Groups

**Figure 1:**
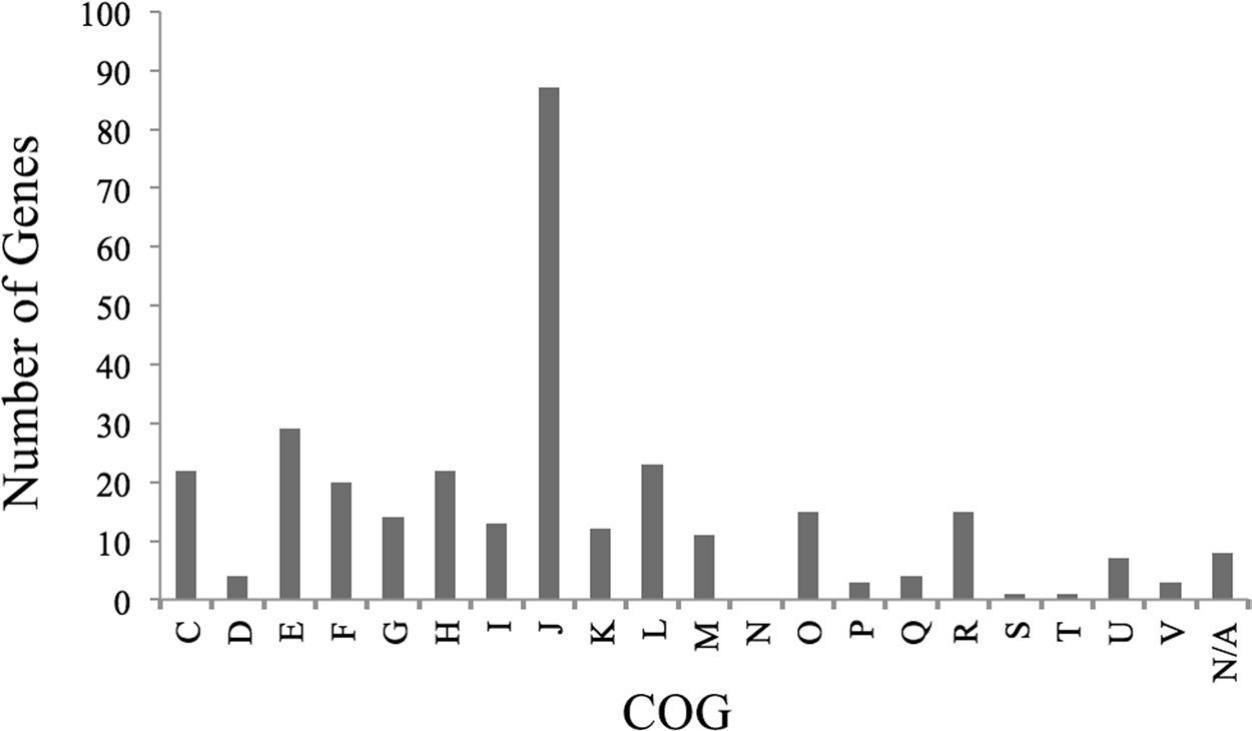
Distribution of functional COGs (Clusters of Orthologous Groups) for the 304 ‘core’ genes analyzed here. Letters refer to COG functional categories as follows. C - Energy production and conversion; D - Cell division and chromosome partitioning; E - Amino acid transport and metabolism; F - Nucleotide transport and metabolism; G - Carbohydrate transport and metabolism; H - Coenzyme metabolism; I - Lipid metabolism; J - Translation, ribosomal structure and biogenesis; K - Transcription; L - DNA replication, recombination and repair; M - Cell envelope biogenesis, outer membrane; N – Cell motility; O - Posttranslational modification, protein turnover, chaperones; P - Inorganic ion transport and metabolism; Q - Secondary metabolites biosynthesis, transport, and catabolism; R - General function prediction only; S - COG of unknown function; T - Signal transduction mechanisms.

**Figure 2:**
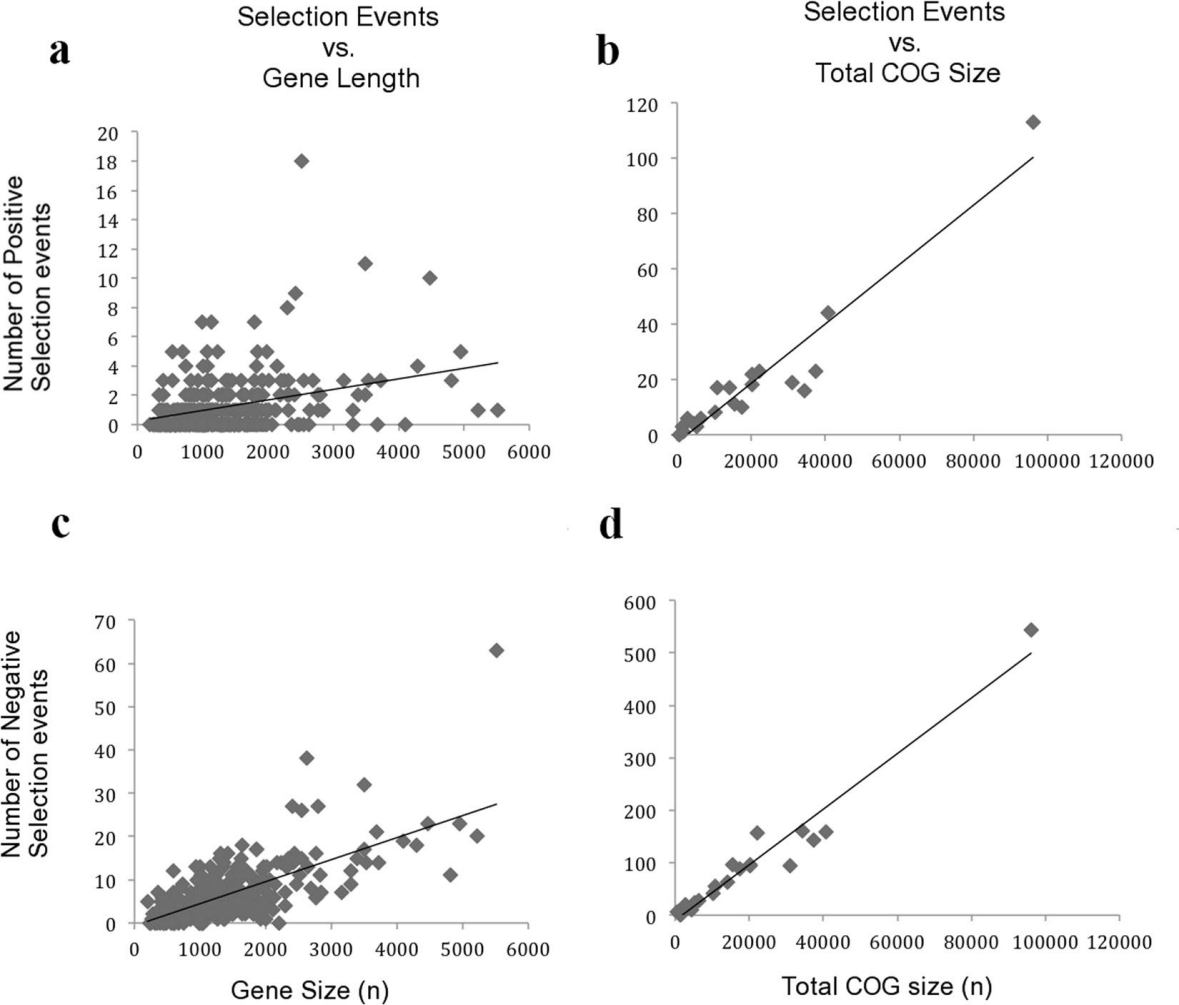
Plots outlining the relationships between number of selection events and gene length or COG size. (**a**) Relationship between gene length and number of nucleotides under positive selection within *Blattabacterium* (F-value: 40.872, Df: 1, p-value: 6.16e-10, adjusted R-squared: 0.12). (**b**) Relationship between total COG size and number of nucleotides under positive selection within *Blattabacterium* (Chi-square p-value: 2.2e-16). (**c**) Relationship between gene length and number of nucleotides under negative selection within *Blattabacterium* (F-value: 189.15, Df: 1, p-value: 2.2e-16, adjusted R-squared: 0.38). (**d**) Relationship between total COG size and number of nucleotides under negative selection within *Blattabacterium* (Chi-square p-value: 2.2e-16).

COG analysis indicates an uneven distribution of functional groups within the 304 genes selected for this analysis (Fig. 1). This figure illustrates the functional ‘core’ genes shared by all thirteen genomes analyzed. The majority of these genes are ribosomal in function. Perhaps unsurprisingly, the number of positive selection events (F-value: 40.872, Df: 1, p-value: 6.16e-10, adjusted R-squared: 0.12) as well as negative selection events (F-value: 189.15, Df: 1, p-value: 2.2e-16, adjusted R-squared: 0.38) both showed strong positive correlation with gene length (Fig. 2a,c, respectively). This finding is consistent with the conclusions of previous studies, where natural selection is also correlated with gene length^83^. Building upon this on a functional level, however, we also noted that signatures of both positive and negative selection (response variable) correlated strongly with the total number of nucleotides assigned to a specific COG (explanatory variable) across the *Blattabacterium* genome (Positive selection events: Chi-square p-value: 2.2e-16; Negative selection events: Chi-square p-value: 2.2e-16; Fig. 2b,d, respectively).

### *Blattabacterium* Selection Analysis

Initial analysis of *Blattabacterium* homolog sets was carried out across all eight of the fully sequenced strains, using a significance level of p ≤ 0.05 for homology. At this significance level, *Blattabacterium* displays a strong negative mutational bias, with a ratio of sites under negative selection to sites under positive selection of 11:1 across 304 genes. While most loci within *Blattabacterium* displayed a bias towards negative selection, a few did exhibit signatures of positive selection (Table 2a). That the vast majority of genes within the *Blattabacterium* genome are experiencing neutral (Table 2b) or negative (not shown in table) selection suggests conserved selective pressures and genome architectures within established endosymbiont lineages^28,69^. Accordingly, only a small number of loci were found to show no signs of selection at all (Table 2c).

**Table 2:** (a) Loci within *Blattabacterium* displaying a positive selection bias.

Position	Locus	Avg. Length (n)	COG	No. of Pos. sites	No. of Neg. sites
**a**					
61	*gyrB*	2513	L	18	11
96	*tatC*	1045	U	2	0
171	*purB*	1843	F	5	4
191	*marC*	742	U	2	1
239	*folE*	861	H	2	1
265	*gmk*	751	F	1	0
266	*rpiB*	606	G	1	0
308	*rplX*	324	J	2	1
312	*rplP*	541	J	5	4
319	*rplC*	821	J	3	2
359	*entC*	1374	Q	3	1
365	*recQ*	2201	LKJ	3	0
387	*accA*	1232	I	5	3
388	*sdhB*	985	C	7	0
392	*phospho*	1985	R	5	3
405	*hinT*	536	FGR	3	2
457	*pdxA*	1362	H	2	1
**b**					
38	*trmE*	1821	R	4	4
161	*accD*	1098	I	1	1
167	*purF*	1997	F	1	1
196	*accB*	626	I	1	1
208	*evoX*	1009	L	2	2
241	*m22*	843	O	1	1
304	*rplF*	712	J	1	1
318	*rplD*	821	J	2	2
430	*rpoD*	1127	K	7	7
438	*integral*	995	P	1	1
478	*glyS*	1910	J	3	3
483	*pth*	772	J	1	1
**c**					
12	*truA*	1001	J	0	0
103	*rpsT*	317	J	0	0
117	*aroK*	674	E	0	0
148	*rpmI*	249	N/A	0	0
149	*rplT*	456	J	0	0
204	*rpmG*	239	J	0	0
240	*nadE*	1028	H	0	0
301	*rplO*	601	J	0	0
329	*rplM*	587	J	0	0
332	*cdsA*	1035	R	0	0
372	*rplL*	487	J	0	0
422	*sufE*	567	R	0	0
428	*rpsO*	347	J	0	0
491	*nfsA*	442	N/A	0	0
**d**					
93	*dapF*	1043	E		
112	*gcvH*	523	E		
148	*rpmI*	249	N/A		
323	*rpsL*	499	J		
398	*rpsU*	267	N/A		

Positive selection is defined as those loci that display a greater number of sites under positive selection than under negative selection. (b) Loci within *Blattabacterium* displaying a neutral selection bias. Neutral selection is defined as those loci which display an equal number of sites under positive selection as negative selection. (c) Loci within *Blattabacterium* displaying no selection. These genes experience neither positive nor negative selection events. (d) Loci within *Blattabacterium* and *Flavobacterium* that display identical selection profiles. These genes display no selection events within either *Blattabacterium* or *Flavobacterium* genomes. ‘Position’ indicates that genes starting position within the *Mastotermes darwineinsis* genome, the model *Blattabacterium* genome used here. Letters refer to COG functional categories as follows. C - Energy production and conversion; D - Cell division and chromosome partitioning; E - Amino acid transport and metabolism; F - Nucleotide transport and metabolism; G - Carbohydrate transport and metabolism; H - Coenzyme metabolism; I - Lipid metabolism; J - Translation, ribosomal structure and biogenesis; K - Transcription; L - DNA replication, recombination and repair; M - Cell envelope biogenesis, outer membrane; N – Cell motility; O - Posttranslational modification, protein turnover, chaperones; P - Inorganic ion transport and metabolism; Q - Secondary metabolites biosynthesis, transport, and catabolism; R - General function prediction only; S - COG of unknown function; T - Signal transduction mechanisms.

In recent years, a growing body of work seeks to place an increased emphasis on the role of selection in molecular evolution^84–86^. While no predominant explanatory theory for molecular evolution has yet emerged to replace the largely disproven neutral theory, a re-evaluation of the classic, primarily neutral/drift-centric hypotheses for genome evolution in *Blattabacterium* is necessitated. With the data presented here - and in the light of previous studies into the genome evolution of *Blattabacteria* - we suggest that the *Blattabacterium* genome is shaped by a combination of random genetic drift, environmental selection, and physiological constraint on genetic variation. The *Blattabacterium* lifestyle is characterized by significant and repeated population bottlenecks with each host generation as bacterial cells are transmitted vertically from mother to offspring^17,18^, a drastically reduced genome^25–30,39^, and elevated rates of mutation. Previous studies into obligate bacterial endosymbiont evolution suggest that the reduction in effective population size through generational bottlenecks and lack of genetic recombination resulting from Muller’s Ratchet elevates the rate of fixation of slightly deleterious mutations through random genetic drift^33–35,41–44^. However, populations that experience a population bottleneck recover much of the lost genetic variation through rapid population growth^45^. While it seems likely that this is the case for free-living and endosymbiotic bacteria as well, the strength of the bottleneck affects the loss of genetic variability much more so than subsequent rates of population growth^45^. Within *Blattabacterium* and many other bacterial endosymbionts, these bottlenecks are not trivial, and are frequently recurring throughout the insect host’s lifespan^1–3,7,8,16^; an environment that is completely atypical for most free-living populations. Thus, examined alone, population bottlenecks strongly reduce the genetic variation of *Blattabacterium*. Additionally, *Blattabacterium* – like other intracellular bacterial endosymbionts - reproduces asexually and lacks genetic recombination [^51–56^ reviewed in ref.^57^]; two mechanisms otherwise crucial for the recovery of genetic variation. This combination of factors – lack of genetic recombination and repeated population bottlenecks – does seem to suggest that *Blattabacterium* and other obligate symbionts are less capable of recovering lost genetic variance after population bottlenecks than free-living bacteria.

However, bacterial endosymbionts also experience much higher mutation rates than their free-living relatives^63,69,70^. Indeed, mutations are synonymous with increased genetic variation, and we show here that *Blattabacterium* experiences highly elevated rates of mutation compared to free-living bacterial populations. It is highly unlikely that the elevated mutation rates seen in *Blattabacterium* are adaptive or somehow function in recovering lost genetic variation, as mutations in *Blattabacterium* show a strong bias towards deletions rather than insertions; a pattern that is in agreement with previous studies as well as with Muller’s Ratchet^44,50^. Additionally, it is suggested that reduced strength of selection on many genes in the endosymbiont genome increases the number of nucleotide sites that may be altered without consequences in fitness, strengthening the impact of deletion biases^44^. Bacterial genomes are primarily functional DNA, and the drastic genome reduction observed within *Blattabacterium* and has come at the cost of physiological functionality. Intriguingly however, this drastic loss in functionality does not yet appear to have strong negative impacts on *Blattabacterium* survival or host fitness. Indeed, many physiological tasks are now taken over by the cockroach host, rendering many *Blattabacterium* genes superfluous within the relatively safe and predictable symbiotic environment^25–30,36^. As in other obligate endosymbionts, many if not most of these genes come under relaxed selection, as their function is critical to neither *Blattabacterium’s* survival nor the symbiotic physiological requirements of its cockroach host^32,35^.

Whether or not elevated mutation rates in physiologically-important genes functions to actively reduce genome size and thus streamline bacterial reproduction, or are the result of random genetic drift is unknown; though that many genes lost by *Blattabacterium* since transitioning to an intracellular lifestyle coded for otherwise critical functionality - including the loss of many genes involved in DNA maintenance and repair [^19,28,30,59^, reviewed in ref.^57^] – suggests that many losses are either only mildly non-adaptive or compensated for by the host and thus do not result in immediate impairment of symbiont or host. However, genome reduction is accompanied by a reduction and cell size and a substantial reduction in energy and nutrients requirements, providing an adaptive payoff for the active removal of non-essential genes. Indeed, a number of prokaryotic *Prochlorococcus* species display adaptive and rapid genome shrinkage, with genomic patterns similar to those observed in obligate symbionts including reduced G + C content, elevated rates of mutation, and the loss of DNA-repair genes^87^. However, despite these similarities, genome reduction in *Prochlorococcus* is characterized by largely *neutral* selection, as large population sizes impose low genetic drift and strong purifying selection^87^. Naturally, if genome reduction in *Blattabacterium* and other bacterial endosymbionts was being driven by adaptive forces and not random genetic drift, then we might expect patterns of selection similar to those in the free-living *Prochlorococcus*. Instead, we find here that the overwhelming majority of mutations in the *Blattabacterium* genome are negative in direction, strongly suggesting that genome reduction is not driven by selective processes, but rather by random genetic drift; as has been suggested for numerous other obligate bacterial endosymbionts^32,35^.

Specific genes within the endosymbiont genome are expected to vary among endosymbiont lineages as a function of the metabolic and physiological requirements of the host species. As such, these species-specific genes vital to bacterial survival and/or host fecundity experienced elevated selective pressures for their persistence within the *Blattabacterium* genome. We suspect that many genes in *Blattabacterium* involved in functions critical to this bacterial-host symbiosis display neutral or positive signatures of selection. Thus, while random genetic drift appears to play a strong role in shaping the *Blattabacterium* genome, physiological constraint acts to maintain *Blattabacterium*’s functionality as a primary nutritional endosymbiont across the cockroach lineage. Accordingly, the *Blattabacterium* genome architecture and composition is the result of the interplay between random genetic drift and the fixation of slightly deleterious mutations on one hand and physiological constraint promoting maintenance of cockroach-required metabolic functionality on the other.

When compared to the signatures of selection and patterns of evolution noted within other obligate bacterial symbionts, *Blattabacterium* shows striking similarity. While the ratio of negative to positive selection sites of 11:1 is specific to *Blattabacterium-Flavobacterium* comparisons, similar patterns of strong negative selection have been observed in other insect endosymbiont genomes^69^. Unsurprisingly then, our results conform to the findings of Brynnel *et al*.^33^, who also measured that the *tuf* gene of *Buchnera* is evolving more than 10 times as quickly than the same gene in the free living *E. coli* and *S. typhimurium*. Additionally, *Blattabacterium* - like *Wigglesworthia* and *Buchnera* – does show some evidence for maintaining those functions that are highly important to its insect host^33,63,66,70^. Indeed, the combined effects of Muller’s Ratchet appears to be ubiquitous within obligate insect bacterial symbionts: the *Buchnera* chaperonin *groEL* displays a 5-fold increase in non-synonymous mutations, and a 10-fold increase in synonymous mutations, when compared to *E. coli*^63^. Mutational pressure alone likely does not account for the magnitude of these d_N_/d_S_ rate elevations. Within *Buchnera*, it has been suggested that this elevation of fixation occurs through random genetic drift resulting from the continual reduction of effective endosymbiont population size with each transmission from host parent to host offspring^33,34,88^. Given that this same elevation of polymorphisms is observed within *Blattabacterium* - and that *Blattabacterium* also undergoes similar population bottlenecks with each host generation - it is likely that similar mechanisms are shaping these two independent lineages. This also parallels the findings of Brynnel *et al*.^33^, whom suggested that the rate of synonymous codon substitution within *Buchnera* can be as much as 40 times higher than its free-living relatives.

### Blattabacterium - Flavobacterium Selection Comparison

**Figure 3:**
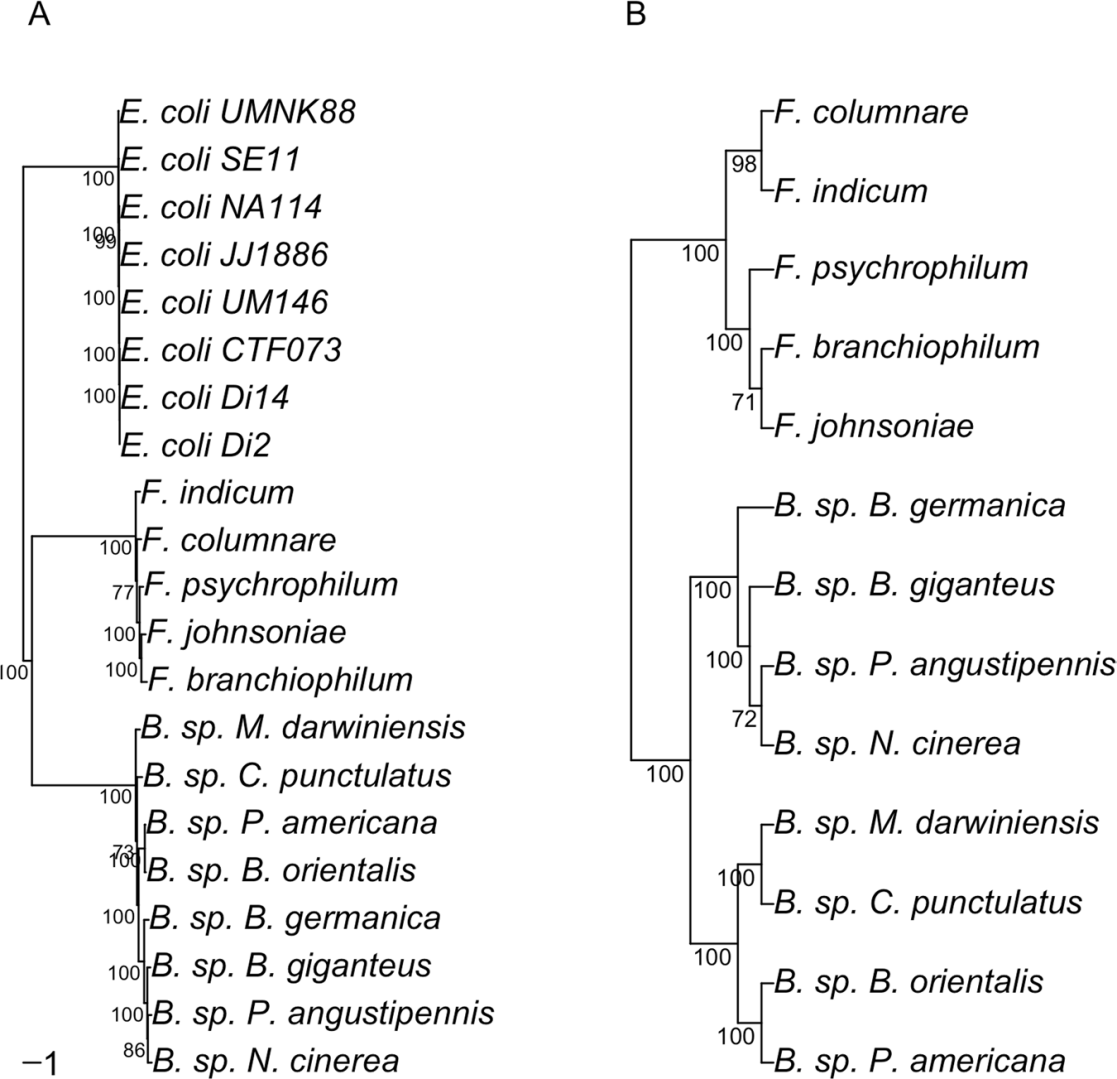
Phylogenetic reconstruction of the evolutionary relationship between all bacteria sampled for this project. (**A**) Maximum likelihood phylogram based on whole genomes from *Flavobacteria* and *Blattabacterium* lineages, with *E. coli* strains as outgroup. Numbers below nodes represent percentage bootstrap support. (**B**) ASTRAL cladogram representing the species tree inferred from 200 nuclear gene trees. Numbers below nodes represent multi-locus bootstrapping support (100 replicates).

*Blattabacterium* displays elevated levels of both positive and negative selection events at a significance level of p ≤ 0.05 when compared to free-living *Flavobacterium*, indicating a genome-wide increase in mutation rates across the examined genes. In order to ensure that these patterns are not the result of sequences displaying radically different divergence times, we performed a phylogenetic analysis (Fig. 3) to elucidate the sequence similarity within each examined group. Phylogenetic analysis of both the *Blattabacterium* group (Table 3) and *Flavobacterium* group (Table 4) indicate similar levels of phylogenetic divergence between the individuals of each^22,89,90^.

**Table 3:** Absolute sequence divergence in the 16S rRNA gene of *Blattabacterium*.

	BPLAN	BCpu	BBge	BGIGA	MADAR	BNCIN	BBor	BPane
BPLAN		0.048	0.037	0.044	0.056	0.04	0.015	0.04
BCpu	0.048		0.038	0.043	0.048	0.043	0.043	0.045
BBge	0.037	0.038		0.024	0.045	0.026	0.038	0.021
BGIGA	0.044	0.043	0.024		0.059	0.026	0.044	0.021
MADAR	0.056	0.048	0.045	0.059		0.049	0.051	0.056
BNCIN	0.04	0.043	0.026	0.026	0.049		0.039	0.021
BBor	0.015	0.043	0.038	0.044	0.051	0.039		0.036
BPane	0.04	0.045	0.021	0.021	0.056	0.021	0.036	

A phylogenetic tree was created using the 16S rRNA gene from each sequenced *Blattabacterium* species. From this tree, phylogenetic distances were calculated in order to estimate sequence similarity and divergence. Host species abbreviations are as follows: BNCIN*, N. cinerea*; BGIGA, *B. giganteus;* BBge, *B. germanica*; BPLAN, *P. americana;* BCpu, *C. punctulatus;* MADAR, *M. darwiniensis*, BBor, *B. orientalis*; BPane, *P. angustipennis spadica*.

**Table 4:** Absolute sequence divergence in the 16S rRNA gene of *Flavobacterium*.

	Fpsych	Fbranch	Fjohn	Findic	Fcolum
Fpsych		0.041	0.053	0.081	0.064
Fbranch	0.041		0.056	0.084	0.068
Fjohn	0.053	0.056		0.086	0.069
Findic	0.081	0.084	0.086		0.063
Fcolum	0.064	0.068	0.069	0.063	

A phylogenetic tree was created using the 16S rRNA gene from each *Flavobacterium* species used in this study. From this tree, phylogenetic distances was estimated. Species abbreviations: Findic, *Flavobacterium indicum*; Fjohn, *Flavobacterium johnsoniae*; Fpsych, *Flavobacterium psychrophilim*; Fbranch, *Flavobacterium branchiophilum*; Fcolum, *Flavobacterium columnare*.

In addition, each group displays comparable percentages of identical sites (*Blattabacterium*: 89.4%, *Flavobacterium*: 87.8%) as well as similar pairwise percent identities (*Blattabacterium*: 95.7%, *Flavobacterium*: 93.3%) when aligning the ribosomal 16S rRNA gene. Thus, extant *Blattabacterium* display signs of elevated rates of genome evolution in the form of increased levels of selection events. The increase in the number of sites experiencing negative or positive selection when compared to the free-living *Flavobacterium* suggests elevated levels of functional protein evolution in the endosymbionts. Only a limited number of loci display similar selection profiles between *Blattabacterium* and *Flavobacterium* (Table 2d).

Results of MEME selection analysis indicate that all genes analyzed show at least some evidence of negative selection. Sites under negative selection comprise approximately 86 percent of examined loci. However, four loci, at one site each, show evidence for positive selection (Table 5). Three of the four loci showing evidence for positive selection are involved in DNA or RNA modification: 2-methylthioadenine synthetase, Holliday Junction resolvase, and 50S ribosomal protein L25 subunit. Within *E. coli* and *Salmonella typhimurium*, variations of the protein 2-methylthioadenosine have been shown to stabilize codon-anticodon interactions through the restriction of first codon position wobble during tRNA aminoacylation^91,92^. This functionality prevents the misreading of the genetic code, thus reducing the likelihood of mutation. Additionally, Holliday Junction resolvase-like proteins have been shown to play key roles in DNA recombination and repair^93–95^. Finally, genes responsible for the production of ribosomes within a cell are crucial for the proper translation of proteins from mRNA^96,97^. Modifications to genes responsible for the production of ribosomal proteins will likely impact the efficiency and/or accuracy of protein translation and assembly. Given the broad reduction in functionality of the *Blattabacterium* genome, and the loss of many ancestral DNA and RNA maintenance and repair genes (Fig. 1)^8,98,99^, it is in some ways not surprising that all currently-described *Blattabacterium* strains display similar selection pressures on those remaining genes responsible for the maintenance of genetic material. However, of notable absence from our list of genes showing signatures of positive selection is the molecular chaperone and maintenance gene *GroEL*. These sequences are part of the larger *GroL* locus in modern *Blattabacterium* genomes, regions of which were found previously in *Blattabacterium* to be under positive selection^99^. This inconsistency likely arises from the outgroups used in each study. We utilized *Blattabacteria’s* closest free-living relative, *Flavobacterium*^16,17^, as an outgroup while Fares *et al*. utilized relatively distantly-related free-living *Gammaproteobacteria*^100^. Based on this methodological distinction, we can conclude that the selective pressure noted by Fares *et al*. was exerted prior to the split between *Blattabacterium* and *Flavobacterium*.

**Table 5:** Loci containing sites that display evidence for positive selection, according to MEME episodic selection analysis. First column denotes the locus of interest. Second column contains the names of the proteins coded by these loci; and the third column contains proposed functional information about these proteins, gathered from the UniProt gene database.

Loci	Protein Name	Putative Function
miaB	2-methylthioadenine synthetase B family tRNA modification enzyme	RNA modification
Holliday Junction	Holliday junction resolvase-like protein	hydrolase, nucleic acid binding, DNA recombination, transcription antitermination
rplY	50S ribosomal protein L25	rRNA binding, negative regulation of translation, translation
atpG	ATP synthase F1 subunit gamma	ATP binding, plasma membrane ATP synthesis coupled proton transport

In contrast to the previous genes, however, which are involved in the maintenance of genetic material, the remaining locus found to show signatures of positive selection, *atpG*, codes for ATP synthase F1 subunit gamma. ATP synthase-family subunit proteins typically combine to form an ATP synthase complex, which is responsible for energy production in the form of ATP within the cell^101,102^. One of the primary functions of *Blattabacterium* within its host is amino acid synthesis. Amino acid production is a very endergonic process, requiring large amounts of energy in the form of ATP in order to effectively carry out biosynthesis^103^. Therefore, beneficial modifications to genes coding for an ATP synthase subunit that result in the more efficient functioning of ATP synthase as a complete complex are more likely to be favored within the *Blattabacterium* genome. In keeping with the previous findings that all *Blattabacterium* strains examined to date are alike in their function to provide essential and nonessential amino acids to their cockroach hosts, here we demonstrate that *Blattabacterium* also share signatures of positive selection within genes responsible for the production of the ATP synthase F1 subunit.

## Conclusions

Our findings indicate that the *Blattabacterium* genome is experiencing elevated rates of both positive and negative selection when compared to its free-living relative *Flavobacterium*, approaching a 10-fold increase in selection rate at the significance level p ≤ 0.05 across 304 individual genes. In combination with previous studies elucidating the evolutionary patterns in other insect endosymbionts, we conclude that the *Blattabacterium* genome is shaped by similar evolutionary mechanisms. Previous studies have outlined the current state of the *Blattabacterium* genome, which is drastically reduced from its ancestral state and possesses a very strong bias towards A + T nucleotide base pairs. Analysis of these trends indicate that *Blattabacterium* are experiencing an accumulation of slightly deleterious mutations through the continued effects of random genetic drift resulting from consecutive population bottlenecks throughout *Blattabacterium’s* evolutionary history, with physiological constraint acting to maintain genes important to bacterial survival and host fecundity. Additionally, *Blattabacterium* has lost many of the genes involved in DNA repair, likely through similar mechanisms discussed here, thus exacerbating this evolutionary bias towards slightly deleterious mutations. That these mutations cannot be repaired increases functional protein evolution rates within this endosymbiont. The patterns discussed here are highly similar to those evolutionary and genomic trends observed in other intracellular insect endosymbionts^34,45,61,90^. Additionally, our analyses also provide insight into the direction of selection of loci within the genome. A vast majority of loci in all *Blattabacterium* genomes analyzed here show signs of negative selection. Only a small fraction of loci (*miaB, Holliday Junction, rplY, atpG*) show signs of positive selection. These observations are in accordance with our previous understanding of the evolutionary history of *Blattabacterium*, as well as its function within its cockroach host as a nutritional endosymbiont aiding in the recycling of nitrogenous waste and the production of both essential and nonessential amino acids.

The analysis presented here could be augmented through a robust analysis of genome reduction within *Blattabacterium*. Using a parsimony approach, the ancestral genome of another primary insect endosymbiont, *Buchnera-Ap*, was reconstructed by Moran and Mira^32^. The results of Moran and Mira’s analysis indicated that much of the ancestral *Bucnhera* genome was lost during a relatively small number of large deletion events shortly after this bacteria’s transition to an intracellular lifestyle. While it is likely that that the *Blattabacterium* genome was reduced through similar mechanisms, a similar reconstruction within this group would offer us a more complete picture of the evolutionary origins of this unique cockroach endosymbiont.

## Data Availability

All data used herein was procured from public NCBI databases; see Table 1.
